# A porcine large animal model of radiofrequency ablation-induced left bundle branch block

**DOI:** 10.3389/fphys.2024.1385277

**Published:** 2024-04-19

**Authors:** Felix Wiedmann, Max Jamros, Valerie Herlt, Amelie Paasche, Manuel Kraft, Moritz Beck, Merten Prüser, Atilla Erkal, Maren Harder, Marcin Zaradzki, Jasmin Soethoff, Matthias Karck, Norbert Frey, Constanze Schmidt

**Affiliations:** ^1^ Department of Cardiology, University Hospital Heidelberg, Heidelberg, Germany; ^2^ DZHK (German Center for Cardiovascular Research), Partner Site Heidelberg/Mannheim, University of Heidelberg, Heidelberg, Germany; ^3^ HCR (Heidelberg Center for Heart Rhythm Disorders), University Hospital Heidelberg, Heidelberg, Germany; ^4^ Department of Cardiac Surgery, University Hospital Heidelberg, Heidelberg, Germany

**Keywords:** cardiac resynchronization therapy, CRT, conduction system pacing, CSP, large animal model, LBBB, left bundle branch block

## Abstract

**Background:**

Electrocardiographic (ECG) features of left bundle branch (LBB) block (LBBB) can be observed in up to 20%–30% of patients suffering from heart failure with reduced ejection fraction. However, predicting which LBBB patients will benefit from cardiac resynchronization therapy (CRT) or conduction system pacing remains challenging. This study aimed to establish a translational model of LBBB to enhance our understanding of its pathophysiology and improve therapeutic approaches.

**Methods:**

Fourteen male pigs underwent radiofrequency catheter ablation of the proximal LBB under fluoroscopy and ECG guidance. Comprehensive clinical assessments (12-lead ECG, bloodsampling, echocardiography, electroanatomical mapping) were conducted before LBBB induction, after 7, and 21 days. Three pigs received CRT pacemakers 7 days after LBB ablation to assess resynchronization feasibility.

**Results:**

Following proximal LBB ablation, ECGs displayed characteristic LBBB features, including QRS widening, slurring in left lateral leads, and QRS axis changes. QRS duration increased from 64.2 ± 4.2 ms to 86.6 ± 12.1 ms, and R wave peak time in V6 extended from 21.3 ± 3.6 ms to 45.7 ± 12.6 ms. Echocardiography confirmed cardiac electromechanical dyssynchrony, with septal flash appearance, prolonged septal-to-posterior-wall motion delay, and extended ventricular electromechanical delays. Electroanatomical mapping revealed a left ventricular breakthrough site shift and significantly prolonged left ventricular activation times. RF-induced LBBB persisted for 3 weeks. CRT reduced QRS duration to 75.9 ± 8.6 ms, demonstrating successful resynchronization.

**Conclusion:**

This porcine model accurately replicates the electrical and electromechanical characteristics of LBBB observed in patients. It provides a practical, cost-effective, and reproducible platform to investigate molecular and translational aspects of cardiac electromechanical dyssynchrony in a controlled and clinically relevant setting.

## 1 Introduction

The necessity to maintain a near-synchronous electrical activation sequence within and between the ventricles of the heart is a key component for preserving normal cardiac pump function (Littmann and Symanski, 2000; Strik et al., 2012; Nguyên et al., 2018). Disturbances of the heart’s synchronized electrical circuitry as they occur with left bundle branch (LBB) block (LBBB) or right ventricular (RV) pacing, lead to mechanical dyssynchrony, which carries the risk of impairing cardiac ejection fraction, thereby posing a risk factor for the development of heart failure (Surkova et al., 2017; Nguyên et al., 2018; McDonagh et al., 2021).

Cardiac resynchronization therapy (CRT), which aims to offset electrical cardiac dyssynchrony by simultaneous or sequential pacing of both ventricles, has shown significant reductions in mortality and morbidity in large clinical trials performed in selected heart failure patients (Bristow et al., 2004; Cleland et al., 2005; Moss et al., 2009; Tang et al., 2010; Zareba et al., 2011). However, the fact that approximately one-third of patients show no evidence of clinical or echocardiographic response after CRT device implantation, and that the optimal left ventricular lead position remains controversial after more than two decades of CRT, highlights the need for further investigation into the effects of cardiac dyssynchrony and resynchronization on cardiac ejection fraction (McDonagh et al., 2021; Glikson et al., 2022; Fyenbo et al., 2023). In addition, in more recent years, a variety of conduction system pacing (CSP) approaches have emerged, in which the pacemaker lead is placed in close proximity to the His bundle or LBB allowing direct stimulation of the cardiac conduction system (Glikson et al., 2022). Despite the rapid clinical adoption of these technologies, there is still limited understanding of their mechanistic basis and impact on cardiovascular physiology (Herweg et al., 2021).

Animal models have been indispensable in uncovering the pathophysiology of electrical and mechanical cardiac dyssynchrony and the beneficial mechanistic effects of CRT (Strik et al., 2012). The first dyssynchronous animal model was established over a century ago, revealing specific changes in QRS morphology through a small incision on the left or right surface of the cardiac septum in a dog’s heart (Eppinger and Rothberger, 1910). Although LBBB has been studied in several species, including sheep, monkeys, and pigs, the canine heart has long been the most widely used animal model to study LBBB (Vernooy et al., 2007; Rigol et al., 2013; Duchenne et al., 2019). The pig model presents itself as a feasible alternative due to its close anatomical and physiological resemblance to humans, as well as being a more economical and ethically accepted model than the dog. However, the porcine model poses certain challenges, notably its vulnerability to ventricular fibrillation during procedures like endocardial ablation (Walcott et al., 2015), due to its transmural Purkinje fiber distribution and its unique coronary venous anatomy (Rigol et al., 2013). This susceptibility has historically impeded the development of a suitable porcine model for studying cardiac dyssynchrony and CRT.

Therefore, establishing a closed-chest model of radiofrequency (RF) ablation-induced LBBB in pigs is a promising avenue to advance research in this field. This study aims to address the disparity between our present understanding of electrical and mechanical cardiac dyssynchrony and the complex patient-tailored understandings required to advance therapeutic methods in the clinical setting. To achieve this, a practical, cost-effective, and replicable LBBB model in domestic pigs was established as an experimental platform to study the molecular and translational characteristics of cardiac electromechanical dyssynchrony in a clinically relevant setting.

## 2 Material and methods

### 2.1 Experimental design, animal handling, and anesthesia

The study was conducted in n = 14 male German Landrace pigs (2–5 months old; body weight 30–45 kg). A total of n = 6 acute experiments were conducted to establish the methodology. In addition, n = 5 animals were followed up for a period of 21 days with daily 6-lead surface ECGs. A group of three further pigs were implanted with a CRT pacemaker (CRT-P) on the seventh day after the LBB ablation and were followed up for another 14 days under CRT. Pigs underwent thorough clinical and electrophysiological characterization, including blood sampling, 12-lead electrocardiography (ECG), echocardiography, electrophysiological studies, and 3D electroanatomical mapping prior to LBBB induction and after a follow-up period of 7 (n = 8) and 21 days (n = 5). The induction of anesthesia was performed as previously described (Schmidt et al., 2019; Wiedmann et al., 2020a; Wiedmann et al., 2020b; Wiedmann et al., 2022). On the first day of the experiment, pigs were sedated with azaperone (5 mg/kg i.m.; Elanco, Bad Homburg, Germany), midazolam (1 mg/kg i.m.; Hameln Pharma Plus GmbH, Hameln, Germany), and ketamine (10 mg/kg i.m.; Zoetis Deutschland GmbH, Berlin, Germany) before anesthesia was induced with propofol (1.5 mg/kg bolus i.v.; followed by 4–8 mg/kg/h i.v.; Zoetis). For analgesia buprenorphine (0.02 mg/kg i.v.; Bayer Vital GmbH Tiergesundheit, Leverkusen, Germany) was administered. Mechanical ventilation was performed using the Draeger Primus system (Draeger, Lübeck, Germany). Prior to gaining vascular access, a single dose of cefuroxime was administered (750 mg i.v.; Ratiopharm GmbH, Ulm, Germany). No volatile anesthetics were used before completion of electrophysiological studies to avoid pharmacological interaction with cardiac ion channels, and anesthesia was initially maintained with propofol (4–8 mg/kg/h i.v.). After completion of electrophysiological studies, anesthesia was continued with isoflurane (0.5–2 vol. %; Baxter Deutschland GmbH, Heidelberg, Germany). Pigs were kept under specific pathogen-free conditions at a room temperature of 20°C ± 2°C with a maximum housing density according to directive 2010/63/EU. Room lighting had a 12/12 h light/dark cycle. Water was offered *ad libitum* and pigs were fed two times a day with balanced complete feed (SAF 130M, ZG Raiffeisen, Karlsruhe, Germany). Environmental enrichment was provided with biting woods, chains, and feeding balls. Porcine hearts were explanted at the end of final surgery after euthanization by i.v. injection of 40 mL potassium chloride (1 mol/L) in deep anesthesia (propofol, isoflurane, buprenorphine). Perioperative antibiotic prophylaxis of 750 mg cefuroxime i.m. and analgesic therapy with buprenorphine i.m. titrated according to effect were continued 48 h after the procedures. The persistence of LBBB was confirmed by daily 6-lead ECGs which were recorded during feeding.

### 2.2 Echocardiography

Echocardiography was performed in spontaneous breathing anesthetized animals. LV systolic function was assessed in modified long-axis, two-, and four-chamber views (Vivid E9, General Electric Company (GE) Germany GmbH, Munich, Germany). To quantify electromechanical dyssynchrony before and after LBBB induction, septal-to-posterior wall motion delay (SPWMD) and LV as well as RV electromechanical delays (LVEMD, RVEMD) were quantified. SPWMD was defined as the delay between the inward deflection of the septal and posterior wall in M-mode (short axis view), LVEMD, and RVEMD as the time from the onset of the QRS complex to the beginning of ventricular ejection as determined by pulsed-wave Doppler in the LV and RV outflow tract, respectively. The interventricular mechanical delay (IVEMD) was calculated as the difference of LVEMD and RVEMD.

### 2.3 Surface ECG

Daily surface ECGs were recorded using a Mortara ELI 230 ECG recorder (Mortara instrument Inc., Milwaukee, WI, United States) with conventional adhesive electrodes (3M red dot, 3M, Maplewood, MN, United States) in the classic Einthoven/Goldberger chest lead configuration during feeding, and QT intervals were corrected using the formula of Bazett (1920).

### 2.4 Intracardiac electrophysiological measurements and 3D electroanatomical mapping

Intracardiac electrophysiological measurements were performed as reported previously (Schmidt et al., 2019; Wiedmann et al., 2020a; Wiedmann et al., 2020b; Wiedmann et al., 2022). Cannulation of the right jugular vein in the anesthetized, mechanically ventilated animal was performed guided by anatomic landmarks. Three venous introducer sheaths (2 × 6 F, 1 × 8 F, Avanti, Cordis Germany GmbH, Norderstedt, Germany) were inserted using the Seldinger technique. Cannulation of the left carotid artery was performed under ultrasound guidance using the micro puncture technique. After establishment of arterial access, systemic anticoagulation with heparin was performed as described below. Under fluoroscopic guidance, quadripolar catheters (JSN 5 F, Abbott Laboratories, Chicago, Il, United States) were placed at the junction of the superior vena cava with the RA and in the RV apex. A UHS 20 stimulus generator (Biotronik, Berlin, Germany) was used for intracardiac pacing and BARD Clearing (Bard Electrophysiology Division, Lowell, MA, United States) was used for recording, analysis, and storage of ECGs. Pacing thresholds ranged from 0.5 V to 3 V at 2.9 ms, and pacing was performed at twice the diastolic pacing threshold. The atrial effective refractory period (AERP) was measured with a conditioning series of nine basic stimuli (S1; 500 ms, 400 ms, or 300 ms, as indicated) followed by an additional stimulus (S2) that started from 70 ms. The coupling intervals of the S2 stimuli were increased in 5 ms steps until the capture of the S2 stimulus was repetitively reached. The atrioventricular node effective refractory period (AVNERP) and RV effective refractory period (RVERP) were measured with a conditioning series of nine basic stimuli (S1; 500 ms or 400 ms as indicated) followed by an additional stimulus (S2) that started 150 ms higher than the expected refractory period and was decreased in 10 ms decrements until capture was repetitively lost. The points of antegrade atrioventricular node Wenckebach periodicity or 2:1 conduction were measured by monitoring the ventricular response under incremental atrial pacing. To measure sinus node recovery time (SNRT), overdrive suppression was performed for 30 s by atrial burst simulation with S1 cycle lengths of 700–300 ms and pre-automatic pauses from the last stimulus to the first intrinsic atrial activation were measured. Corrected sinus node recovery times (cSNRT) were calculated by subtracting the intrinsic cycle length from the respective SNRT. Electroanatomical mapping of the RV and LV was performed before and after LBBB induction using the EnsiteVelocity system (St. Jude Medical, Little Canada, MN, United States). Activation maps were obtained by manually collecting 49–97 points with a steerable 7 F ablation catheter (Biosense Webster, Inc. a Johnson & Johnson company, Diamond Bar, California, United States). A steerable quadripolar catheter (Xtrem, ELA Medical, Montrouge, France) placed in the coronary sinus was used as an intracardiac reference.

### 2.5 RF ablation of the proximal LBB

RF catheter ablation of the proximal LBB was guided by fluoroscopy and intracardiac signals. To prevent induction of ventricular fibrillation during RF ablation, pigs were subjected to high-frequency pacing 10 bpm below the Wenckebach point via the RA catheter during RF ablation. Immediately before ablation, amiodarone (600 mg) and magnesium (6 mmol magnesium aspartate hydrochloride 3-H2O) were administered intravenously. If ventricular fibrillation was induced, the pigs were defibrillated by applying a 200 J biphasic shock through adhesive defibrillation paddles connected to an external defibrillator (M-Series XL, ZOLL Medical Corporation, Chelmsford, Massachusetts, United States). To prevent systemic thromboembolism, the arterial sheaths were continuously flushed with heparinized sodium chloride (0.9% solution, 1,000 IU heparin/L). After placement of the arterial sheaths, pigs received an initial bolus of 5,000 IU heparin (i.v.) and an additional 500 IU every 30 min until the end of the procedure. A steerable non-irrigated 7 F 4-mm tip ablation catheter with a D-curve (Biosense Webster), connected to an RF generator (SmartAblate, Stockert, Freiburg, Germany), was advanced into the RA through the previously placed venous introducer sheath and guided to the His bundle by fluoroscopy and intracardiac signals. This catheter position was radiographically recorded (40° right anterior oblique (RAO) angulation on the supine pig). The catheter was then inserted through the arterial sheath and advanced retrogradely into the LV. The catheter was retracted from the apex towards the basal LV outflow tract below the aortic valve until it projected approximately onto the previously marked position of the His bundle. If a His signal was detected at the distal pole pair of the catheter, the catheter tip was, again, advanced distally toward the apex while continuously monitoring intracardiac signals until an LBB potential (distance to QRS complex onset <40 ms) was registered at the distal pole pair. After localization of the ablation site, RF ablation was performed at 30 W. If a sudden change in morphology, axis, and duration of the surface ECG (QS in V1, R in V6, widening of the QRS complex, and T-wave inversion) was observed, RF energy delivery was continued for 30 s. If atrioventricular conduction disturbances occurred during ablation, energy delivery was immediately stopped, and the catheter position was adjusted toward the apex. After successful ablation of the proximal LBB, the induced LBBB was monitored on the surface ECG for 15 min. If the typical ECG changes persisted over this period, the endpoint of LBBB induction was reached.

### 2.6 CRT pacemaker implantation

Animals receiving CRT stimulation were anesthetized on day 7 as described above. After creating an intramuscular pocket in the region of the lateral neck muscles, venous access was obtained via the jugular vein using the Seldinger technique. Active fixation pacemaker leads (CapSureFix 5076, Medtronic, Dublin, Ireland) of 52 cm and 58 cm length were placed in the RA appendage and the apicoseptal region of the RV under radiographic control using two 7 F splittable introducers sheaths (Abbott). After stimulation threshold (<1 V at 0.4 ms), impedance (300–1,000 Ω), and sensing (>1.5 mV in the atrium and >5 mV in the ventricle) were confirmed by the PSA function of the Abbott Merlin pacemaker interrogator, pacemaker leads were sewed to the muscular fascia with three 0-gauge braided non-absorbable sutures each. Following a medial thoracotomy via hemisternotomy, the pericardium was opened. A unipolar epimyocardial pacing lead of 50 cm (Medtronic, CAPSURE EPI 4965) was sutured to the posterolateral LV (2–0 monofilament non-absorbable suture) after mapping the site of latest activation by repeated repositioning of the lead. For this purpose, the distance from the beginning of the Q wave in the surface ECG to the signal of the LV electrode (Q-LV delay) was determined by the PSA function of the Abbott Merlin pacemaker interrogator. Following sternal osteosynthesis using wire cerclage, the LV lead was tunneled into the pacemaker pocket. After connecting all probes to a Medtronic Syncra CRT-P device, careful hemostasis, antibiotic irrigation of the device pocket with cefuroxime, and layered wound closure were performed.

### 2.7 Statistical analysis

Surface ECGs obtained during the interventions and follow-up period were digitized and analyzed using ImageJ software (National Institutes of Health, Bethesda, MD). Intracardiac ECGs were measured and displayed via the BARD EP Lab system (Boston Scientific Corporation, Marlborough, Massachusetts, United States). Analysis of mapping data was conducted using the Ensite Velocity workstation (St. Jude Medical). Statistical analysis and data visualization were conducted with Prism 9.5 (GraphPad Software, La Jolla, CA, United States). The data are presented as mean ± standard deviation (SD). Paired or unpaired two-tailed Student's t-tests were used to determine statistical significance of two groups and the probability values were adjusted for multiple comparisons using the Bonferroni correction unless otherwise specified. In case of small sample sizes normality was assessed using Shapiro-Wilk tests. For comparisons of three or more groups one-way repeated measures ANOVA were employed. Where significant effects were detected, *post hoc* analyses were conducted using Tukey’s Honestly Significant Difference test. A *p*-value of less than 0.05 was considered statistically significant.

## 3 Results

### 3.1 Experimental procedure and RF ablation of the proximal LBB in pigs

A total of n = 6 acute experiments were conducted to establish the methodology. In addition, n = 5 animals were followed up for a period of 21 days with daily 6-lead surface ECGs. The feasibility of CRT was further tested by equipping n = 3 animals with a CRT-P device 1 week post LBBB induction, which were then followed up for a further 2 weeks under CRT. 12-lead surface ECGs, blood sampling and echocardiography were performed under baseline conditions, on day 7 and day 21. Electrophysiological studies and endocardial 3D electroanatomical mapping studies were performed on day 0 before and after ablation as well as on day 21.

**FIGURE 1:**
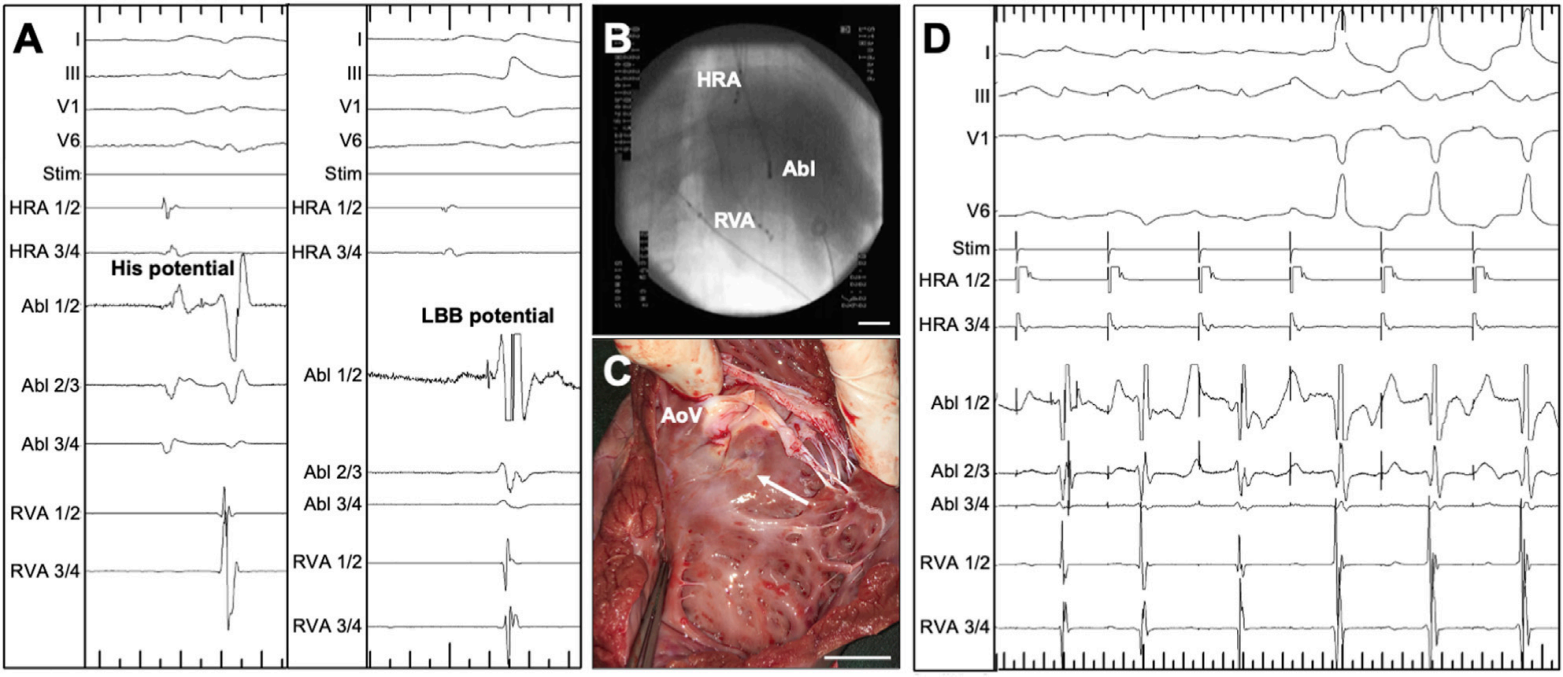
Radiofrequency (RF) ablation of the proximal left bundle branch (LBB) in pigs, guided by intracardiac ECG and X-ray. **(A)** Intracardiac and surface ECG recordings, visualizing the signal on the ablation catheter tip (Abl 1/2) at the position of the His bundle (left, His potential) and at the position of the LBB (right, LBB potential). Major ticks indicate 100 ms. **(B)** Intraoperative fluoroscopy, showing the position of the ablation catheter (Abl) catheter as well as quadripolar diagnostic catheters in the high right atrium (HRA) and right ventricular apex (RVA). **(C)** View of the left ventricular aspect of the interventricular septum of an explanted porcine heart. The circumscribed RF ablation lesion is located somewhat below the cusps of the aortic valve (AoV). **(D)** ECG-documentation of the typical response upon RF-ablation of the LBB: Under antiarrhythmic atrial pacing, an intrinsic atrioventricular nodal conduction with initially narrow QRS complexes can be seen. The spike of the LBB potential is visible in the channel of the tip of the ablation catheter. After the start of RF ablation, the axis of the QRS complex changes within seconds and the characteristic QRS widening can be seen, which is particularly evident in the leftward leads I and V6. Major ticks indicate 1,000 ms. Stim, monitor of the stimulation channel.

Intracardiac signal and fluoroscopy-guided RF ablation of the proximal LBB was performed as described above (compare Figure 1A for the intracardiac ECG signals, Figure 1B for the intraprocedural fluoroscopy). A circumscribed RF ablation lesion was observed on the LV side of the basal interventricular septum during organ removal as part of the final procedure (Figure 1C). The appearance of characteristic changes in ECG morphology prompted continuous RF energy delivery for 30 s (Figure 1D). After successful ablation of the proximal LBB, the induced LBBB was monitored on the surface ECG for 15 min. If the typical ECG changes persisted over this period, the endpoint of LBBB induction was reached.

In this study, an average of five ablations per animal were required to establish persistent LBBB, with a range of 1–14. The mean incidence of ventricular fibrillation episodes induced by RF ablation was 2 (range 0–7). A notable observation was the apparent improvement in the proficiency of the interventionalist over the course of the study. Initially, the first five animals required an average of nine ablation attempts, inducing three episodes of ventricular fibrillation. Conversely, for the final quintet, the averages dropped to three ablations and 2 VF episodes per pig, demonstrating a clear learning curve. One of 14 animals (7.1%) showed a transient AV block that transitioned to a second-degree AVB within a few minutes and then resolved completely over time. Daily clinical assessments showed no evidence of heart failure after LBBB induction. In addition, blood sampling did not reveal any significant abnormalities on day 7 or day 21 (Supplementary Tables S1–S2).

### 3.2 Clinical and surface ECG characteristics of the porcine LBBB model

**FIGURE 2:**
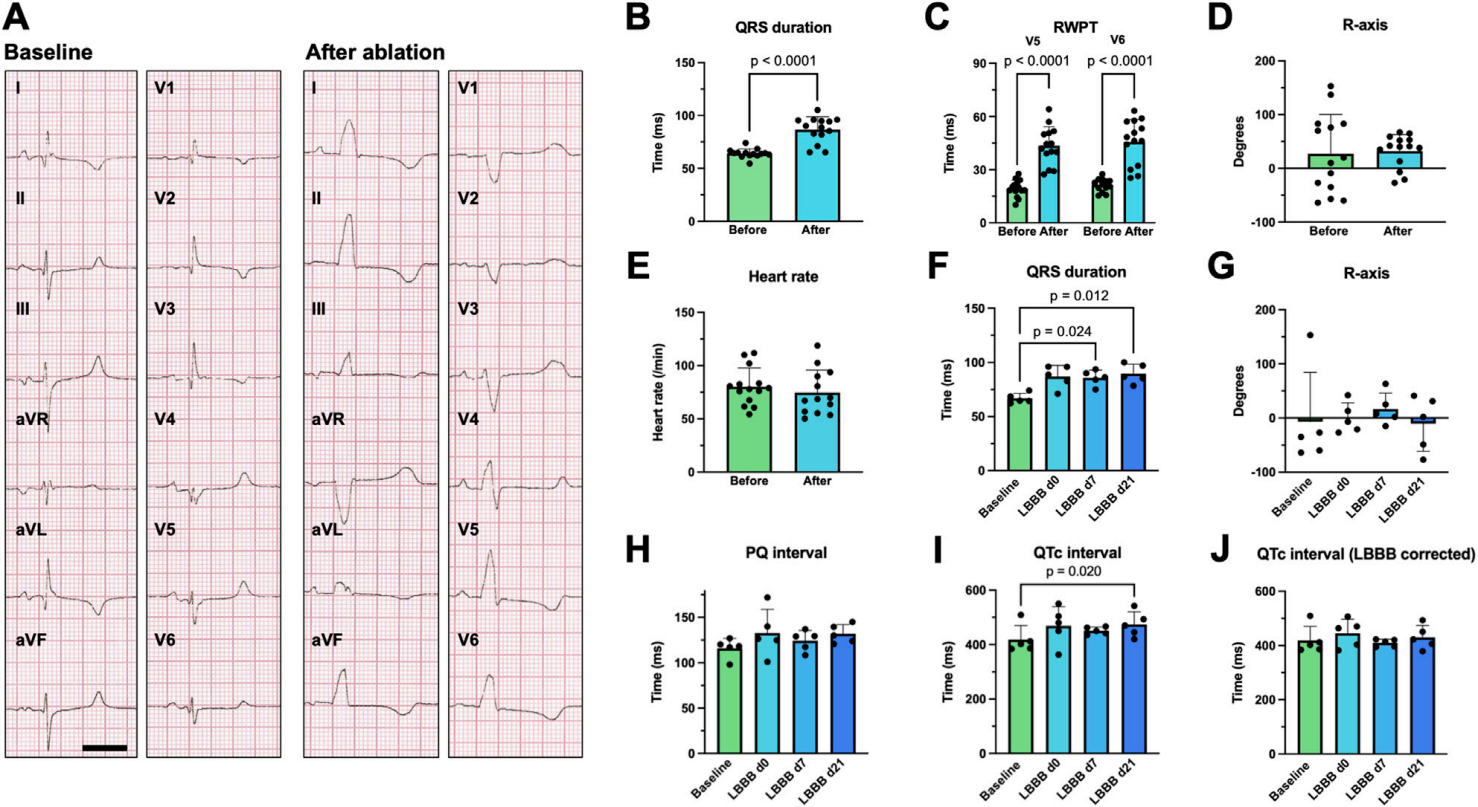
Surface ECG changes following radiofrequency (RF) ablation of the proximal left bundle branch (LBB). **(A)** Example 12-lead surface ECGs recorded on a supine, anesthetized pig with electrodes placed in standard positions before (baseline) and after RF ablation of the proximal LBB. The scalebar depicts 200 ms. **(B–D)** Acute change in QRS duration, R wave peak time (RWPT) and R-axis upon RF ablation of the proximal LBB (n = 14 pigs). **(E)** Heart rate of the pigs before and after RF-ablation. **(F–J)** Time course of the development of the QRS duration, R-axis, PQ interval, QTc interval as well as QTc interval corrected for LBB-block (LBBB) using the method, published by Bogossian et al. (2014) (n = 5). Data are given as mean ± standard deviation. *p*-values, derived from paired two-tailed Student's t tests, followed by Bonferroni correction or from one-way repeated measures ANOVAs in the case of three or more groups are depicted as insets.

Representative 12-lead surface ECG recordings obtained before (baseline) and after LBB ablation from an anesthetized pig in supine position are shown in Figure 2A. A significant widening of the QRS complex upon LBBB induction can be observed, particularly in leads I, aVL, and V5, V6. Here, a slight slurring of the QRS is also evident. Accordingly, in the right precordial leads V1 and V2 a deep S-peak is visible and in the left lateral leads ST-segment deviations occur. Interestingly, the characteristic notching of the QRS complex observed in humans was not fully present with the absolute widening of the QRS complex also being less pronounced: Immediately after LBB ablation, the QRS duration increased from 64.2 ± 4.2 ms to 86.6 ± 12.1 ms (n = 14; *p* < 0.0001; Figure 2B). The R-wave peak time (RWPT) measured in V5 or V6 was prolonged from 19.3 ± 4.6 ms to 43.5 ± 10.6 ms (n = 14; *p* < 0.0001; Figure 2C) and from 21.3 ± 3.5 ms to 45.7 ± 12.6 ms (n = 14; *p* < 0.0001; Figure 2C), respectively. Similarly, the R-wave axis, which exhibits significant variability in the supine positioned pig due to the sagittal axis of the heart, was measured at 32.4 ± 30.8° after ablation in Figure 2D, while the heart rate of the pigs did not change significantly (Figure 2E). Our data further show that QRS prolongation persisted over the observation period of 7 and 21 days (Figure 2F), which was also the case for the other ECG parameters (Figures 2G–J). There was a small trend towards prolongation of the PQ interval after ablation, which did not reach statistical significance (n = 5, *p* = 0.35; Figure 2H). On day 21, the QTc interval was significantly prolonged (Figure 2I). This difference was, however, blunted after correcting the QTc interval for the LBBB pattern using the method proposed by Bogossian et al. (2014) (Figure 2J).

### 3.3 Mechanical signs of dyssynchrony in the porcine LBBB model

**FIGURE 3:**
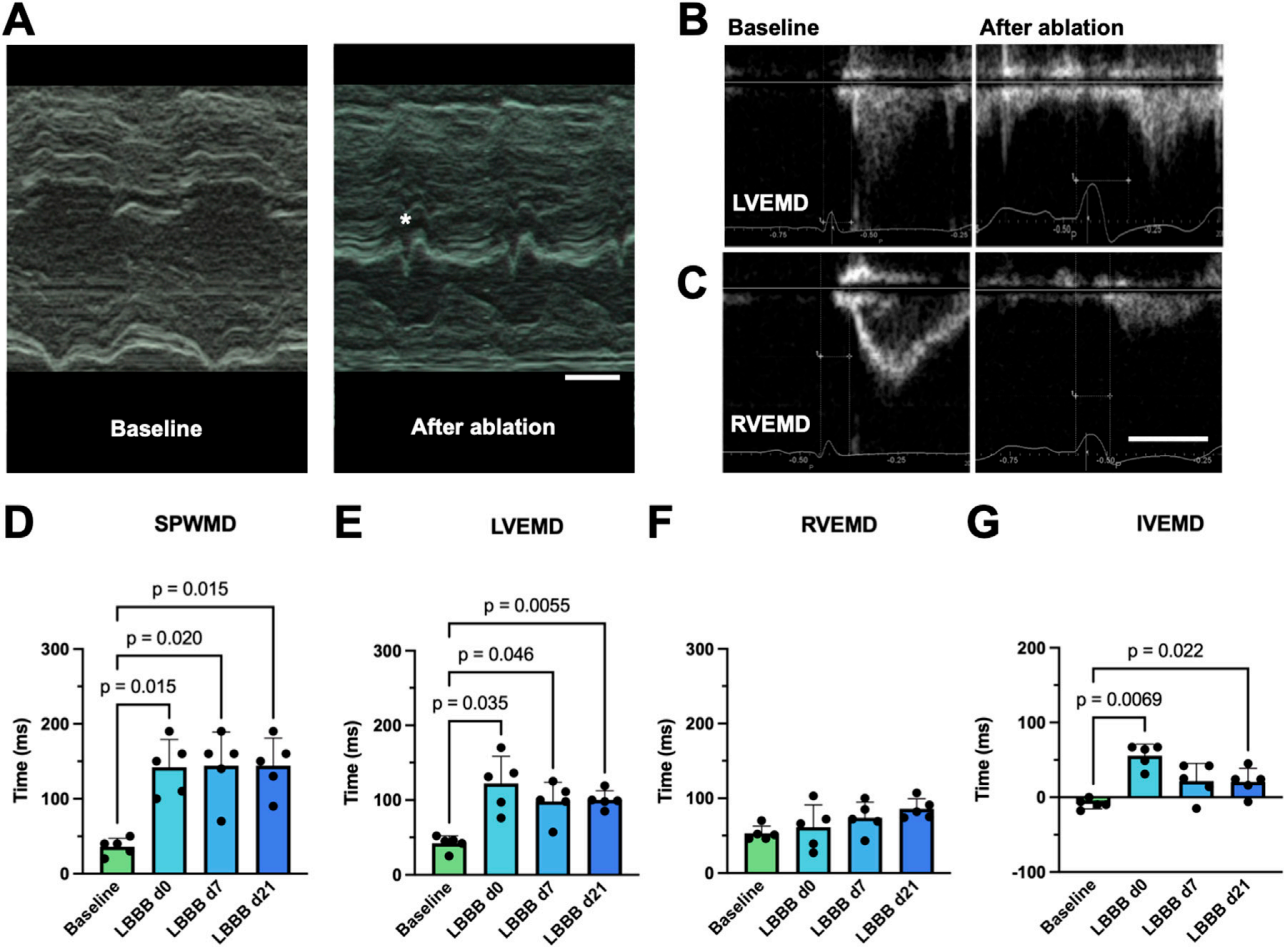
Echocardiographic features of radiofrequency (RF) ablation-induced left bundle branch (LBB) block (LBBB). **(A)** Representative M-mode tracings, obtained from a parasternal short axis view of the left ventricle before (left, baseline) and after RF ablation of the proximal LBB. Upon LBBB induction a delay between contraction of the septal and posterior wall as well as a characteristic “septal flash” (*) can be observed. The scalebar depicted bottom right indicates 200 ms **(B–C)** Echocardiographic determination of the left- **(B)** and right- **(C)** ventricular electromechanical delay (LVEMD/RVEMD). A pulsed-wave Doppler was positioned at either the left or right ventricular outflow tract from a long-axis view, and the time interval between the onset of the QRS complex in lead I of the parallel recorded ECG and the initiation of the flow profile curve was determined. The scalebar depicted bottom right indicates 200 ms. **(D)** Quantification of the septal to posterior wall motion delay (SPWMD), determined as the delay between the onset of contraction of the respective wall segments in a setting described under **(A)**. **(E–F)** Quantification of LVEMD and RVEMD among the experimental groups. **(G)** Quantification of the interventricular mechanical delay (IVEMD) as the difference of LVEMD and RVEMD. Data of n = 5 individual pigs are given as mean ± standard deviation. *p*-values, derived from paired two-tailed Student's t tests followed by Bonferroni correction or from one-way repeated measures ANOVAs in the case of three or more groups are depicted as insets.

To evaluate the impact of LBBB induction on mechanical cardiac dyssynchrony, transthoracic echocardiography was conducted before and after LBB ablation, as well as on day 7 and day 21, respectively. Although the LV ejection fraction of the animals remained unchanged (LVEF_d0_ 60.1% ± 5.3%; LVEF_d21_ 60.6% ± 11.1%; n = 5; *p* = 0.88), distinct echocardiographic signs of dyssynchrony, such as the characteristic septal flash (Figure 3A), were apparent. For echocardiographic quantification of cardiac dyssynchrony, SPWMD (Figure 3A) and the delays between onset of electrical and mechanical activation (Figures 3B, C) of both ventricles were analyzed. LBBB induction resulted in a significant prolongation of the SPWMD by 106.0 ± 41.6 ms (n = 5; *p* < 0.015; Figure 3D) that persisted for 7 and 21 days. Moreover, an increase in LVEMD by 80.0 ± 39.6 ms (n = 5; *p* < 0.035; Figure 3E) was observed. Although LVEMD decreased over time, it was still significantly prolonged at day 21. In the context of an unchanged RVEMD, the IVEMD was also significantly prolonged by 64.4 ± 20.1 ms (n = 5; *p* < 0.0069; Figures 3F, G). Thus, echocardiography reflected both intra- and interventricular dyssynchrony.

### 3.4 Electrophysiological effects of altered LV excitation patterns

**FIGURE 4:**
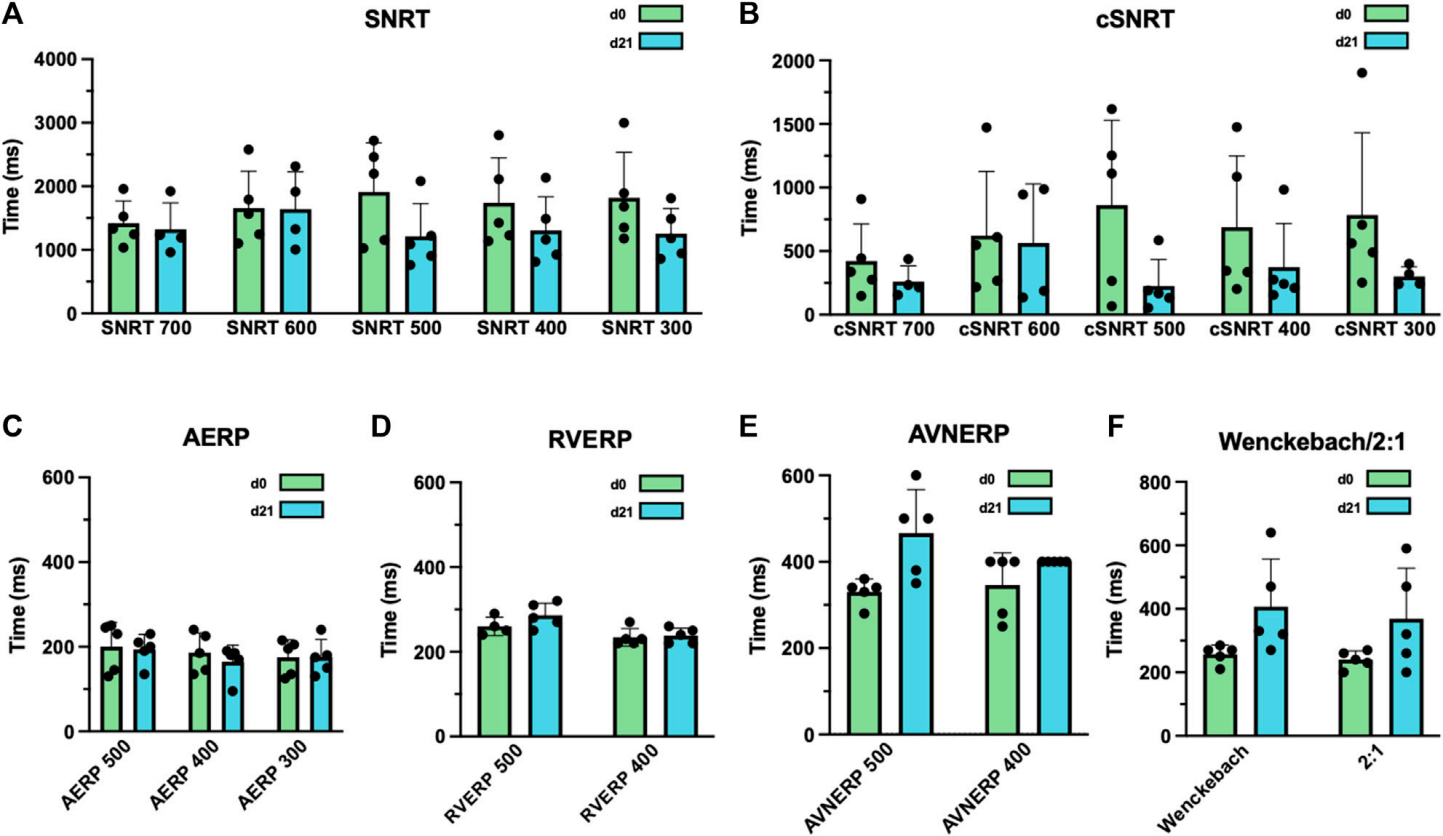
Effects of radiofrequency (RF) ablation-induced left bundle branch (LBB) block (LBBB) on atrial and ventricular electrophysiology. **(A)** Sinus node recovery times (SNRT) were measured after 30 s of atrial overdrive suppression at a pacing cycle length of 300–700 ms (as indicated) before and after LBBB induction. **(B)** Corrected SNRTs (cSNRT) calculated as SNRT minus the respective cycle length of sinus rhythm before and after LBBB induction. **(C)** Atrial effective refractory periods (AERP), measured at an S1 cycle length of 300–500 ms (as indicated) before and after ablation of the proximal LBB. **(D)** Right ventricular effective refractory periods (RVERP), measured at a S1 cycle length of 400 and 500 ms (as indicated) before and after LBBB induction. **(E)** Atrioventricular nodal effective refractory periods (AVNERP), measured at a S1 cycle length of 400 and 500 ms (as indicated) before and after LBBB induction. **(F)** Point of atrioventricular-nodal Wenckebach periodicity or 2:1 conduction before and after ablation of the proximal LBB. Data are given as mean ± standard deviation. No statistically significant differences in paired two-tailed Student's t tests followed by Bonferroni correction, were observed in this experiment.

**FIGURE 5:**
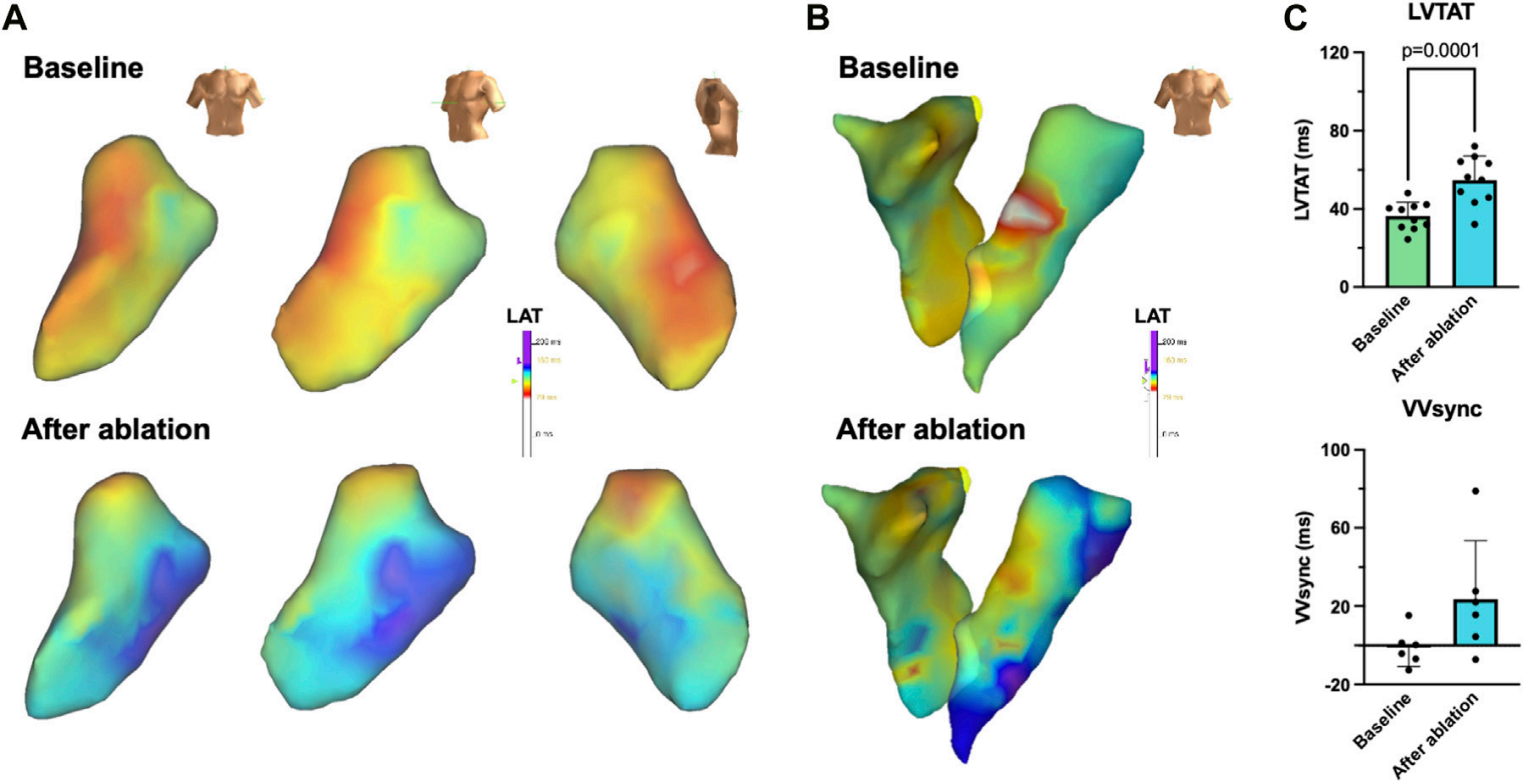
3D electroanatomical mapping of the radiofrequency (RF) ablation-induced left bundle branch (LBB) block (LBBB). **(A)** Representative endocardial 3D electroanatomical activation maps of the left ventricle before (baseline, top) and after RF ablation of the proximal LBB from an anterior-posterior (left) left anterior oblique (mid) or lateral (right) perspective. **(B)** Representative endocardial biventricular activation maps of right and left ventricle before (baseline, top) and after (bottom) LBBB induction. **(C)** Quantification of left ventricular total activation times (LVTAT), defined as the average of the maximum 10% of activation times minus the average of the minimum 10% of activation times (n = 10) as well as the interventricular electrical synchronicity (VVsync), calculated as the difference of the LVTAT and the respective right ventricular total activation times (n = 6). Scalebars are provided as insets. Data are given as mean ± standard deviation. *p*-values, derived from two-tailed Student's t tests followed by Bonferroni correction, are provided.

Electrophysiologic studies performed in anesthetized animals on day 0 prior to LBBB ablation and on day 21 revealed that SNRTs and cSNRTs were not significantly altered upon LBBB induction (Figures 4A, B). The same applied to AERPs and RVERPs as depicted in Figures 4C, D. A trend that did not reach statistical significance was observed for prolongation of AVNERP as well as the points for Wenckebach periodicity and 2:1 conduction of the atrioventricular node upon LBBB induction (Figures 4E, F). The 3D electroanatomical endocardial activation mapping studies which were conducted before and after induction of LBBB revealed a shift of the LV breakthrough site and a subsequently altered pattern of LV excitation with delayed activation of the basolateral and inferolateral wall segments (Figures 5A, B). After induction of LBBB, the LV total activation times (LVTAT), defined as the average of the maximum 10% of activation times minus the average of the minimum 10% of activation times, increased from 36.3 ± 7.1 ms to 54.8 ± 12.3 ms (Figure 5C). The total activation times of the RV (RVTAT) remained unaffected at 38.7 ± 9.8 ms (n = 6; *p* = 0.079). The interventricular electrical synchronicity (VVsync), assessed as the difference of the mean left and right ventricular activation times, shifted from 1.1 ± 9.5 ms to 23.6 ± 29.8 ms (n = 6; *p* = 0.11; Figure 5C).

### 3.5 CRT pilot approach

**FIGURE 6:**
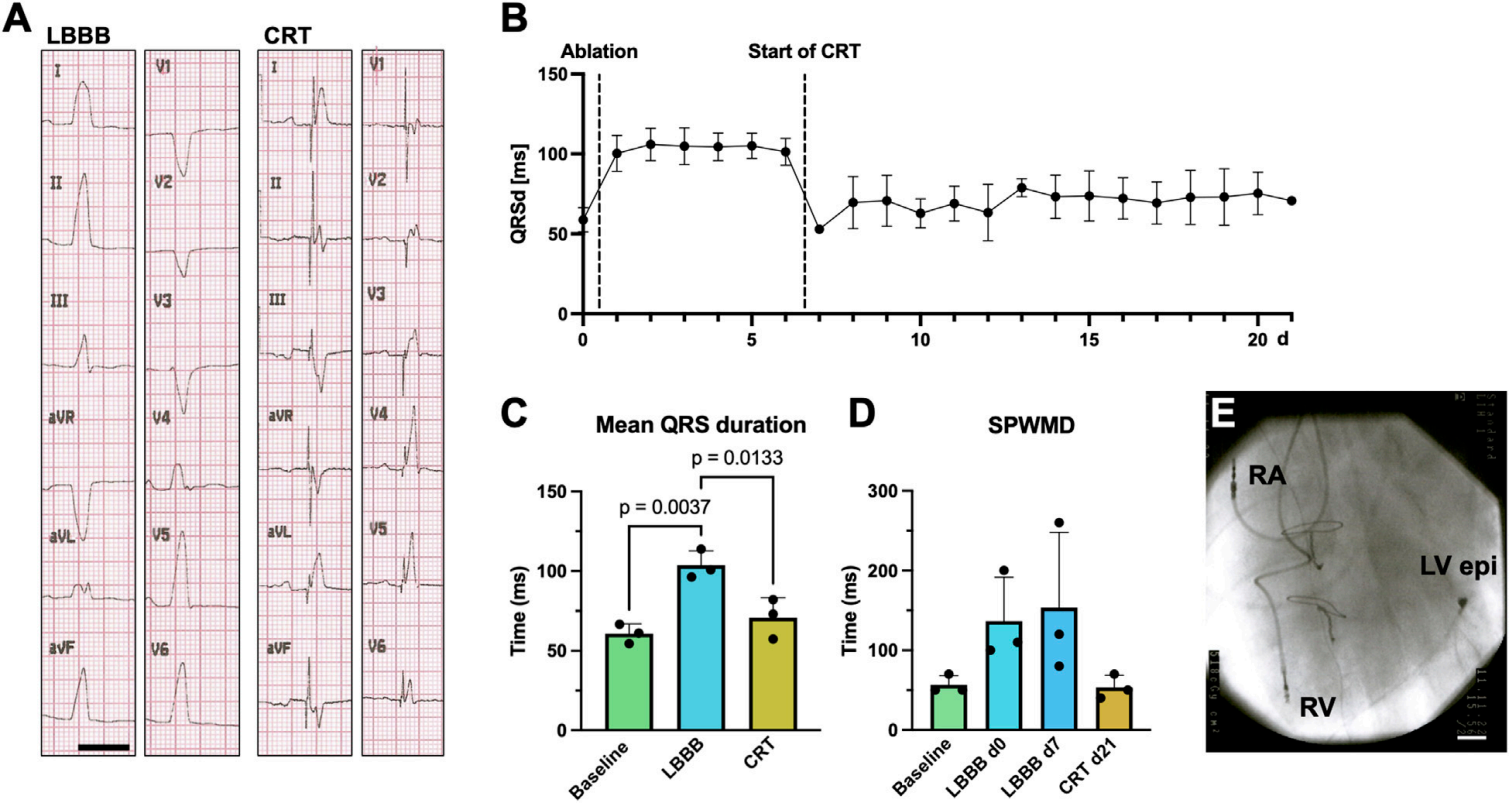
Pilot approach of cardiac resynchronization therapy (CRT) for RF-induced porcine left bundle branch block (LBBB). **(A)** Example 12-lead surface ECGs recorded on a supine, anesthetized pig with electrodes placed in standard positions after LBBB ablation with inactivated (left, LBBB) and activated CRT pacemaker (CRT-P) stimulation (right, CRT). Scalebar depicts 200 ms. **(B)** Time course of QRS duration (QRSd) in daily recorded 6-lead surface ECGs under baseline conditions, upon LBBB induction and CRT-pacemaker stimulation after day 7. **(C)** Quantification of QRS duration on perioperative 12-lead ECGs under baseline conditions, after LBBB induction and upon CRT-stimulation. **(D)** Septal-to-posterior wall motion delays (SPWMD), as determined by echocardiography at the respective time points. **(E)** Postoperative chest X-ray showing the right atrial (RA), right ventricular (RV) and epimyocardial left ventricular (LV epi) electrodes of the CRT-P system. Data of n = 3 individual pigs are given as mean ± standard deviation. *p*-values, derived from one-way repeated measures ANOVAs are depicted as insets.

As the coronary venous anatomy of young landrace pigs is usually not suitable for transvenous placement of a coronary sinus lead, CRT-P systemes with an LV epimyocardial lead were implanted after identification of the site of latest LV activation. Example 12-lead ECG recordings of a supine anesthetized pig with and without CRT are shown in Figure 6A. A significant attenuation of the QRS duration can be observed. Figure 6B shows the QRS duration determined in the daily 6-channel ECGs under baseline conditions, after LBB ablation, and subsequent CRT-P implantation, after which the QRS duration is significantly reduced. On average, the QRS duration increased from 60.8 ± 6.1 ms to 99.9 ± 8.4 ms after LBBB induction (n = 3; *p* = 0.0037; Figure 6C) and was shortened to 75.8 ± 8.8 ms after 14 days of CRT (n = 3; *p* = 0.013; Figure 6C). Successful mechanical resynchronization after CRT-P implantation was demonstrated by a clear trend towards reduction in SPWMD from 153.3 ± 94.5 ms to 53.3 ± 15.3 ms (n = 3; *p* = 0.32; Figure 6D), as well as a trend towards reduction in LVEMD (Supplementary Figure S1). The postoperative chest radiograph after CRT-P implantation is shown in Figure 6E.

## 4 Discussion

The complex interplay between LBBB, cardiac dyssynchrony, and heart failure has been thoroughly studied (Cleland et al., 2005; Moss et al., 2009; Tang et al., 2010). However, it remains unclear why cardiac dyssynchrony leads to the development of LV dysfunction only in certain patients and why only approximately two-thirds of these patients respond to CRT. While canine models have historically provided invaluable insights due to their comparable extent of electrical dyssynchrony to humans, the ethical and financial implications of these models necessitate the exploration of potential alternatives, such as the porcine model (Houser et al., 2012; Strik et al., 2012). Thus, in this study, a practical, cost-effective, and replicable model of LBBB in Landrace pigs was successfully established using RF ablation. The procedure was meticulously designed to minimize complications such as ventricular arrhythmias, thromboembolism, and atrioventricular block. Therefore, it involved precise localization and ablation of the proximal LBB and was monitored through detailed echocardiographic and electrocardiographic follow-up assessments.

The results demonstrate that the model closely reproduces abnormalities found in patients with electrical and mechanical cardiac dyssynchrony. The QRS duration increased from 64.2 ± 4.2 ms to 86.6 ± 12.1 ms (n = 14; *p* < 0.0001), which is consistent with previous reports that QRS duration increases by approximately 50% in pigs undergoing LBBB induction or RV pacing (Marrouche et al., 2002; Strik et al., 2012; Rigol et al., 2013). Additionally, a substantial prolongation of the RWPT was observed in the left precordial leads. Surface ECG pattern concordant with LBBB persisted over a 21-day follow-up period. Echocardiographic indicators of cardiac mechanical dyssynchrony, such as SPWMD, LVEMD, and IVEMD also persisted over the 21-day period, demonstrating the reliability and stability of the model. Changes in ventricular excitation propagation were further traced by 3D electroanatomical activation mapping, which showed a prolongation of LVTAT and a shift of the LV breakthrough site. Additionally, the study included a pilot approach to CRT that demonstrated effective electrical and mechanical resynchronization in the animals as evidenced by reversal of electrocardiographic and echocardiographic parameters of mechanical dyssynchrony. This showcased the model’s potential for translating advanced interventional therapy strategies into clinical practice.

The proposed approach for RF ablation of the proximal LBB is relatively fast and simple to perform and does not require any special interventional skills or electrophysiological knowledge. The ablation of the proximal LBB could have also been performed with the use of a 3D mapping guided RF ablation procedure. However, when establishing this model, this was explicitly avoided to make the procedure less expensive and not to tie it to the availability of special equipment. Therefore, this model can be implemented anywhere anesthesia, X-ray, intracardiac signal visualization, and RF generator are available.

Animal models of LBB have been employed for over a century (Eppinger and Rothberger, 1910). The most commonly used species were dogs, but also sheep and primates, and, in some cases, pigs were employed to study LBBB (Strik et al., 2012; Rigol et al., 2013). Typically used open-chest models have the disadvantage that thoracotomy significantly affects cardiac mechanics and electrophysiological properties (Strik et al., 2012). While catheter-based closed-chest approaches are preferable in this respect, they associated with the difficulty that 1) systematic thromboembolism may occur during ablation, that 2) ventricular tachycardia may arise during catheter manipulation or ablation, particularly in the porcine model (Walcott et al., 2015), and that 3) third-degree atrioventricular block can occur during ablation near the His bundle. In this study, the risk of thromboembolism could be addressed with a simple and effective heparinization protocol, as described above. Based on previously published data, heparin was applied at fixed time intervals, which simplifies the procedure and does not require expensive equipment for measuring the activated clotting time (Rigol et al., 2013). Prophylactic treatment with magnesium and amiodarone was employed to prevent the development of ventricular arrhythmias. In addition, based on previous publications, transient high-rate pacing was performed during ablation, which homogenizes heart rate and ventricular repolarization and suppresses ablation-induced extrasystoles, thus preventing proarrhythmogenic short-long-short sequences (Rigol et al., 2013). In contrast to previous reports, in this study tachystimulation was delivered via the right atrial catheter and the rate was adjusted according to the endogenous atrioventricular conduction capacity, which has the following advantages: 1) Immediate feedback on the success of the ablation. As soon as the QRS morphology change (Figure 1D) occurred, the ablation was continued for 30 s at 30 W, which led to a persistent LBBB in 100% of cases. 2) The ablation can be immediately interrupted in the event of atrioventricular conduction block, which significantly reduces the risk of a third-degree atrioventricular block during bundle-branch ablation in close proximity to the His bundle. The relatively simple conventional ablation approach described in this work also helps to avoid the occurrence of mechanically induced LBBB due to catheter manipulation, which can occur during extensive LV mapping. Mechanically induced LBBB can significantly complicate the localization of the correct ablation site and the assessment of when the endpoint of successful LBB ablation has been reached. This issue was addressed by interrupting the mapping study when relevant QRS morphology changes occurred, ensuring ECG normalization which normally occurred within 10–50 min. We restricted the mapping of the LBB region to very careful catheter movements that were performed immediately prior to ablation. To our knowledge, there are only two other published reports of porcine LBBB models induced by RF ablation (Rigol et al., 2013; Heckman et al., 2020) and one ischemia-induced model (Jorge et al., 2019). As these studies are acute experiments, to our knowledge, this is the first study to describe the chronic induction and therapy of LBBB in a porcine model. The approach proposed in this study represents a refinement since RF ablation of the proximal LBB in the study by Rigol et al. was associated with a 10%–67% risk of developing AVB III° in different study groups and Heckman et al. reported the development of AVB III° in 50% of cases, whereas in the present study high-grade AV block occurred only transiently and was found to resolve completely in the short term (Rigol et al., 2013; Heckman et al., 2020). Of note, while Rigol et al. report having used an irrigated tip ablation catheter, the LBB ablations in this study were performed with a conventional non-irrigated tip ablation catheter. This is expected to produce smaller and more superficial lesions than the use of irrigation, which cools the tip-tissue interface, preventing coagulum formation and allowing higher power settings for longer durations to create deeper tissue lesions. Non-irrigated tip ablation may therefore be beneficial in ablating the very superficial LBB system and sparing the more deeply located structures of the His bundle.

In previous work, the complex procedure of LBB ablation has occasionally been circumvented by performing studies of electrical and mechanical dyssynchrony in large animal models under continuous RV pacing, where electrical stimulation also spreads from the RV to the LV (Spragg et al., 2003; Strik et al., 2012). However, this has been shown to disrupt RV activation because pacing induces slow intramyocardial conduction rather than fast conduction through Purkinje fibers, and the site of pacing-induced septal breakthrough is different from the site of intrinsic conduction breakthrough (Strik et al., 2012).

It must be acknowledged that the criteria proposed for diagnosing a complete typical LBBB in humans were not met in pigs which should be taken into account as a limitation. Only a slight slurring, rather than a broad notch, was observed in leads I, aVL, V5 or V6, and the QRS duration of the bundle branch pattern was on average 86.6 ± 12.1 ms, instead of 135–150 ms, as proposed by Strauss et al. and the current societies’ guidelines (Strauss and Selvester, 2009; McDonagh et al., 2021; Glikson et al., 2022). This may be due to the specific anatomy of the Purkinje fiber system, which is distributed subendocardially in humans but transmurally in pigs (Sedmera and Gourdie, 2014; Elbrønd et al., 2023). It therefore must be taken into account that the hemodynamic consequences of LBBB and its treatment by CSP may differ from the situation in the human heart. When using the model to study methods in the field of CSP, it is important to consider these anatomical differences. Moreover, the mechanism of a locoregional RF ablation is not directly transferable to the mechanisms that take place in patients with, for example, progressive fibrosis of the entire conduction system. However, the echocardiographic examinations show an increase in mechanical dyssynchrony comparable to that of LBBB patients (Gorcsan et al., 2008).

The model used in this study involves surgical placement of an epicardial lead and epicardial mapping to identify late activation sites on the free lateral wall of the LV. This approach differs significantly from the standard clinical practice of placing the left ventricular lead in a branch of the coronary sinus. This discrepancy is significant because a patient’s individual venous anatomy may limit lead placement to only one or two sites, which our epicardial mapping-guided LV lead placement may not accurately reproduce. As the high incidence of CRT non-responders may be partly caused by challenges in pinpointing the latest activation areas within the coronary venous system and the potential lack of suitable veins (McDonagh et al., 2021; Glikson et al., 2022), it is important to consider such a limitation when translating findings from this preclinical model to the clinical setting.

The electrophysiological examinations were carried out without performing a pharmacological complete autonomic blockade. Instead, great efforts were made to standardize the animal handling conditions and the induction of anesthesia in order to keep the activation level of the autonomic nervous system as stable and comparable as possible.

Animal ethics considerations suggest that an experimental project should be conducted in “lower developed” vertebrates whenever possible. However, murine models lack comparability with respect to many electrophysiological properties (Wiedmann et al., 2018). Large animals offer the advantage that they have a comparable size of cardiovascular organs to humans, especially when device- or catheter-based therapies are being evaluated. Landrace pigs, as used in this project, further have the advantage of being affordable and easily accessible. However, their rapid growth limits the experimental phase to usually less than 6 months. Nevertheless, the described technique can also be applied to minipigs, which can be used in longer-term experiments or to investigate questions relating to a different age group. Furthermore, this model allows for the investigation of mechanistic questions by utilizing currently available transgenic and knockout large animal models (Rogers, 2016).

After inducing LBBB, the young and healthy animals studied in this work did not exhibit any signs of heart failure according to clinical, biomarker or echocardiographic findings. If the aim of the planned study would be to evaluate the impact of dyssynchrony on heart failure, an additional stressor must be introduced by, for example, elevating the heart rate through atrial stimulation, as previously detailed for other LBBB animal models (Spragg et al., 2003; Barth et al., 2009). This allows, for example, hemodynamic studies to be performed in acute or chronic experiments, with or without CRT/CSP treatment. Several large animal models, like burst-pacing-induced atrial fibrillation (Schmidt et al., 2019; Wiedmann et al., 2020b; Wiedmann et al., 2022) and pressure-overload models such as stent-based percutaneous aortic constriction procedure (pTAC) (Hinkel et al., 2021), are available for pig studies. These models can be combined with LBB ablation for even more comprehensive studies. Therefore, this model could potentially be used to study the impact of various comorbidities and pathologies on the pathophysiology of dyssynchrony-induced reduction in LV function and the efficacy of CRT/CSP, thereby providing in-depth insights into patient-specific determinants that may influence treatment outcomes.

Taken together, this study successfully established a practical, cost-effective, and replicable large animal LBBB model using RF ablation of the proximal LBB. The model demonstrated significant and consistent electrocardiographic as well as echocardiographic signs of mechanical dyssynchrony over a 21-day period, closely mirroring human LBBB characteristics. Despite limitations in QRS morphology and -duration due to the pigs’ unique conduction system anatomy, the induced mechanical delay was consistent with human LBBB observations. The study’s approach, characterized by its simplicity, reproducibility, and minimal procedural risks, represents a significant advancement in animal models for cardiovascular research. It not only provides a viable alternative to traditional models but also a robust platform for exploring cardiac dysfunctions, the mechanisms of heart failure, and translating new therapeutic interventions in the fields of CRT and CSP into clinics.

## Figures and Tables

**Table:** 

**AERP**	atrial effective refractory period
**ALT**	alanine aminotransferase
**AP**	alkaline phosphatase
**AST**	aspartate aminotransferase
**AVNERP**	atrioventricular node effective refractory period
**CRP**	C-reactive protein
**CRT-P**	cardiac resynchronization therapy pacemaker
**CRT**	cardiac resynchronization therapy
**cSNRT**	corrected sinus node recovery time
**CSP**	conduction system pacing
**ECG**	electrocardiography
**GGT**	gamma-glutamyl transferase
**Hb**	hemoglobin
**HCT**	hematocrit
**HRA**	high right atrium
**LBB**	left bundle branch
**INR**	international normalized ratio
**LBBB**	left bundle branch block
**LV**	left ventricle
**LDH**	lactate dehydrogenase
**LVEMD**	left ventricular electromechanical delay
**LVTAT**	left ventricular total activation time
**MCH**	mean corpuscular hemoglobin
**MCHC**	mean corpuscular hemoglobin concentration
**MCV**	mean corpuscular volume
**n.m**	not measurable
**PLT**	platelets
**pTAC**	percutaneous aortic constriction
**PTT**	prothrombin time test
**QRSd**	QRS duration
**RAO**	right anterior oblique
**RBC**	red blood count
**RF**	radiofrequency
**RVA**	right ventricular apex
**RVEMD**	right ventricular electromechanical delay
**RVERP**	right ventricular effective refractory period
**RVTAT**	right ventricular total activation time
**SD**	standard deviation
**SNRT**	sinus node recovery time
**SPWMD**	septal-to-posterior wall motion delay
**VVsync**	interventricular electrical synchronicity
**WBC**	white blood count

## Data Availability

The original contributions presented in the study are included in the article/Supplementary Material, further inquiries can be directed to the corresponding author.

## References

[B1] Barth A. S. Aiba T. Halperin V. DiSilvestre D. Chakir K. Colantuoni C. (2009). Cardiac resynchronization therapy corrects dyssynchrony-induced regional gene expression changes on a genomic level. Circ. Cardiovasc Genet. 2 (4), 371–378. 10.1161/circgenetics.108.832345 20031609 PMC2801868

[B2] Bazett H. C. (1920). An analysis of the time relations of electrocardiograms. Heart 7, 353–370. 10.1111/j.1542-474X.1997.tb00325.x

[B3] Bogossian H. Frommeyer G. Ninios I. Hasan F. Nguyen Q. S. Karosiene Z. (2014). New formula for evaluation of the QT interval in patients with left bundle branch block. Heart rhythm. 11 (12), 2273–2277. 10.1016/j.hrthm.2014.08.026 25149024

[B4] Bristow M. R. Saxon L. A. Boehmer J. Krueger S. Kass D. A. De Marco T. (2004). Cardiac-resynchronization therapy with or without an implantable defibrillator in advanced chronic heart failure. N. Engl. J. Med. 350 (21), 2140–2150. 10.1056/NEJMoa032423 15152059

[B5] Cleland J. G. Daubert J. C. Erdmann E. Freemantle N. Gras D. Kappenberger L. (2005). The effect of cardiac resynchronization on morbidity and mortality in heart failure. N. Engl. J. Med. 352 (15), 1539–1549. 10.1056/NEJMoa050496 15753115

[B6] Duchenne J. Claus P. Pagourelias E. D. Mada R. O. Van Puyvelde J. Vunckx K. (2019). Sheep can be used as animal model of regional myocardial remodeling and controllable work. Cardiol. J. 26 (4), 375–384. 10.5603/CJ.a2018.0007 29570208 PMC8084358

[B7] Elbrønd V. S. Thomsen M. B. Isaksen J. L. Lunde E. D. Vincenti S. Wang T. (2023). Intramural Purkinje fibers facilitate rapid ventricular activation in the equine heart. Acta Physiol. (Oxf) 237 (3), e13925. 10.1111/apha.13925 36606541

[B8] Eppinger P.-D. D. Rothberger J. (1910). Ueber die Folgen der Durchschneidung der Tawaraschen Schenkel des Reizleitungssystems. Z. für Klin. Med. 70, 1.

[B9] Fyenbo D. B. Bjerre H. L. Frausing M. Stephansen C. Sommer A. Borgquist R. (2023). Targeted left ventricular lead positioning to the site of latest activation in cardiac resynchronization therapy: a systematic review and meta-analysis. Europace 25 (9), euad267. 10.1093/europace/euad267 37695316 PMC10507669

[B10] Glikson M. Nielsen J. C. Kronborg M. B. Michowitz Y. Auricchio A. Barbash I. M. (2022). 2021 ESC Guidelines on cardiac pacing and cardiac resynchronization therapy. Europace 24 (1), 71–164. 10.1093/europace/euab232 34455427 PMC13179788

[B11] Gorcsan J. Abraham T. Agler D. A. Bax J. J. Derumeaux G. Grimm R. A. (2008). Echocardiography for cardiac resynchronization therapy: recommendations for performance and reporting--a report from the American society of echocardiography dyssynchrony writing group endorsed by the heart rhythm society. J. Am. Soc. Echocardiogr. 21 (3), 191–213. 10.1016/j.echo.2008.01.003 18314047

[B12] Heckman L. I. B. Kuiper M. Anselme F. Ziglio F. Shan N. Jung M. (2020). Evaluating multisite pacing strategies in cardiac resynchronization therapy in the preclinical setting. Heart rhythm. 1 (2), 111–119. 10.1016/j.hroo.2020.03.003 PMC8183878 34113865

[B13] Herweg B. Welter-Frost A. Vijayaraman P. (2021). The evolution of cardiac resynchronization therapy and an introduction to conduction system pacing: a conceptual review. EP Eur. 23 (4), 496–510. 10.1093/europace/euaa264 33247913

[B14] Hinkel R. Batkai S. Bähr A. Bozoglu T. Straub S. Borchert T. (2021). AntimiR-132 attenuates myocardial hypertrophy in an animal model of percutaneous aortic constriction. J. Am. Coll. Cardiol. 77 (23), 2923–2935. 10.1016/j.jacc.2021.04.028 34112319

[B15] Houser S. R. Margulies K. B. Murphy A. M. Spinale F. G. Francis G. S. Prabhu S. D. (2012). Animal models of heart failure: a scientific statement from the American Heart Association. Circ. Res. 111 (1), 131–150. 10.1161/RES.0b013e3182582523 22595296

[B16] Jorge E. Solé-González E. Amorós-Figueras G. Arzamendi D. Guerra J. M. Millán X. (2019). Influence of left bundle branch block on the electrocardiographic changes induced by acute coronary artery occlusion of distinct location and duration. Front. Physiol. 10, 82. 10.3389/fphys.2019.00082 30809155 PMC6379473

[B17] Littmann L. Symanski J. D. (2000). Hemodynamic implications of left bundle branch block. J. Electrocardiol. 33, 115–121. 10.1054/jelc.2000.20330 11265710

[B18] Marrouche N. F. Pavia S. V. Zhuang S. Kim Y. J. Tabata T. Wallick D. (2002). Nonexcitatory stimulus delivery improves left ventricular function in hearts with left bundle branch block. J. Cardiovasc Electrophysiol. 13 (7), 691–695. 10.1046/j.1540-8167.2002.00691.x 12139294

[B19] McDonagh T. A. Metra M. Adamo M. Gardner R. S. Baumbach A. Böhm M. (2021). 2021 ESC Guidelines for the diagnosis and treatment of acute and chronic heart failure. Eur. Heart J. 42 (36), 3599–3726. 10.1093/eurheartj/ehab368 34447992

[B20] Moss A. J. Hall W. J. Cannom D. S. Klein H. Brown M. W. Daubert J. P. (2009). Cardiac-resynchronization therapy for the prevention of heart-failure events. N. Engl. J. Med. 361 (14), 1329–1338. 10.1056/NEJMoa0906431 19723701

[B21] Nguyên U. C. Verzaal N. J. van Nieuwenhoven F. A. Vernooy K. Prinzen F. W. (2018). Pathobiology of cardiac dyssynchrony and resynchronization therapy. Europace 20 (12), 1898–1909. 10.1093/europace/euy035 29771311

[B22] Rigol M. Solanes N. Fernandez-Armenta J. Silva E. Doltra A. Duchateau N. (2013). Development of a swine model of left bundle branch block for experimental studies of cardiac resynchronization therapy. J. Cardiovasc Transl. Res. 6 (4), 616–622. 10.1007/s12265-013-9464-1 23636845

[B23] Rogers C. S. (2016). Engineering large animal species to model human diseases. Curr. Protoc. Hum. Genet. 90, 15. 10.1002/cphg.18 PMC4957131 27367161

[B24] Schmidt C. Wiedmann F. Beyersdorf C. Zhao Z. El-Battrawy I. Lan H. (2019). Genetic ablation of TASK-1 (tandem of P domains in a weak inward rectifying K^+^ channel-related acid-sensitive K^+^ channel-1) (K_2P_3.1) K^+^ channels suppresses atrial fibrillation and prevents electrical remodeling. Circ. Arrhythm. Electrophysiol. 12 (9), e007465. 10.1161/circep.119.007465 31514528

[B25] Sedmera D. Gourdie R. G. (2014). Why do we have Purkinje fibers deep in our heart? Physiol. Res. 63 (1), S9–S18. 10.33549/physiolres.932686 24564668

[B26] Spragg D. D. Leclercq C. Loghmani M. Faris O. P. Tunin R. S. DiSilvestre D. (2003). Regional alterations in protein expression in the dyssynchronous failing heart. Circulation 108 (8), 929–932. 10.1161/01.Cir.0000088782.99568.Ca 12925451

[B27] Strauss D. G. Selvester R. H. (2009). The QRS complex-a biomarker that "images" the heart: QRS scores to quantify myocardial scar in the presence of normal and abnormal ventricular conduction. J. Electrocardiol. 42 (1), 85–96. 10.1016/j.jelectrocard.2008.07.011 18790501

[B28] Strik M. van Middendorp L. B. Vernooy K. (2012). Animal models of dyssynchrony. J. Cardiovasc Transl. Res. 5 (2), 135–145. 10.1007/s12265-011-9336-5 22130900 PMC3306020

[B29] Surkova E. Badano L. P. Bellu R. Aruta P. Sambugaro F. Romeo G. (2017). Left bundle branch block: from cardiac mechanics to clinical and diagnostic challenges. Ep Eur. 19 (8), 1251–1271. 10.1093/europace/eux061 28444180

[B30] Tang A. S. Wells G. A. Talajic M. Arnold M. O. Sheldon R. Connolly S. (2010). Cardiac-resynchronization therapy for mild-to-moderate heart failure. N. Engl. J. Med. 363 (25), 2385–2395. 10.1056/NEJMoa1009540 21073365

[B31] Vernooy K. Cornelussen R. N. Verbeek X. A. Vanagt W. Y. van Hunnik A. Kuiper M. (2007). Cardiac resynchronization therapy cures dyssynchronopathy in canine left bundle-branch block hearts. Eur. Heart J. 28 (17), 2148–2155. 10.1093/eurheartj/ehm207 17611254

[B32] Walcott G. P. Kroll M. W. Ideker R. E. (2015). Ventricular fibrillation: are swine a sensitive species? J. Interv. Card. Electrophysiol. 42 (2), 83–89. 10.1007/s10840-014-9964-1 25591724

[B33] Wiedmann F. Beyersdorf C. Zhou X. Büscher A. Kraft M. Nietfeld J. (2020a). Pharmacologic TWIK-related acid-sensitive K^+^ channel (TASK-1) potassium channel inhibitor A293 facilitates acute cardioversion of paroxysmal atrial fibrillation in a porcine large animal model. J. Am. Heart Assoc. 9 (10), e015751. 10.1161/jaha.119.015751 32390491 PMC7660874

[B34] Wiedmann F. Beyersdorf C. Zhou X. B. Kraft M. Foerster K. I. El-Battrawy I. (2020b). The experimental TASK-1 potassium channel inhibitor A293 can Be employed for rhythm control of persistent atrial fibrillation in a translational large animal model. Front. Physiol. 11, 629421. 10.3389/fphys.2020.629421 33551849 PMC7858671

[B35] Wiedmann F. Beyersdorf C. Zhou X. B. Kraft M. Paasche A. Jávorszky N. (2022). Treatment of atrial fibrillation with doxapram: TASK-1 potassium channel inhibition as a novel pharmacological strategy. Cardiovasc Res. 118 (7), 1728–1741. 10.1093/cvr/cvab177 34028533

[B36] Wiedmann F. Schulte J. S. Gomes B. Zafeiriou M. P. Ratte A. Rathjens F. (2018). Atrial fibrillation and heart failure-associated remodeling of two-pore-domain potassium (K(2P)) channels in murine disease models: focus on TASK-1. Basic Res. Cardiol. 113 (4), 27. 10.1007/s00395-018-0687-9 29881975

[B37] Zareba W. Klein H. Cygankiewicz I. Hall W. J. McNitt S. Brown M. (2011). Effectiveness of cardiac resynchronization therapy by QRS morphology in the multicenter automatic defibrillator implantation trial–cardiac resynchronization therapy (MADIT-CRT). Circulation 123 (10), 1061–1072. 10.1161/CIRCULATIONAHA.110.960898 21357819

