# Supplementary material for: A porcine large animal model of radiofrequency ablation-induced left bundle branch block
Source: Front Physiol. 2024 Apr 19;15:1385277. doi: 10.3389/fphys.2024.1385277 (PMC11066324; doi:10.3389/fphys.2024.1385277)
Supplement: Supplementary file 1 [file DataSheet1.PDF]

Supplementary Material

1 Supplementary Figures and Tables

1.1 Supplementary Figures

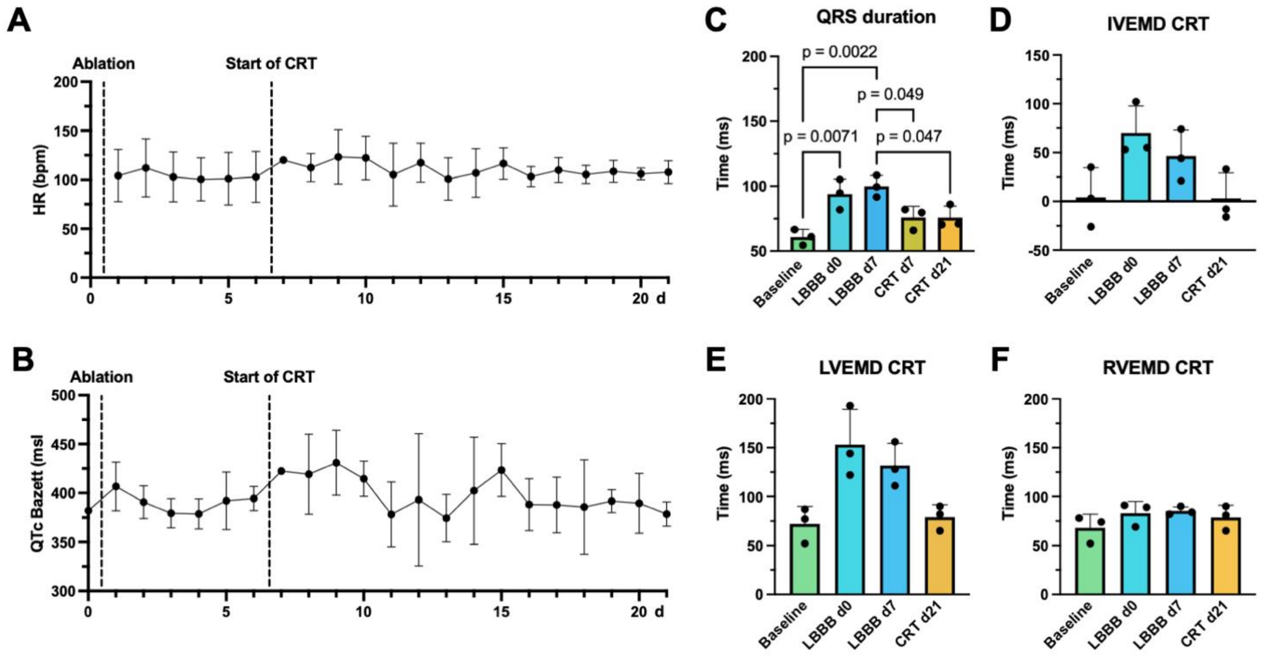

Figure S1: In-depth electrocardiographic and echocardiographic characterization of cardiac resynchronization therapy (CRT) in the radiofrequency (RF) ablation-induced porcine left bundle branch block (LBBB) model. (A) Heart rate (HR) of the animals in daily 6-lead surface ECGs. (B) QT-interval corrected for HR using the formula of Bazett (QTc) of the animals in daily 6-lead surface ECGs. (C) QRS duration, (D) interventricular electromechanical delays (IVEMD), (E) left ventricular electromechanical delays (LVEMD) and (F) right ventricular electromechanical delays (RVEMD) under baseline conditions, following LBBB induction and upon CRT. Data of n = 3 individual pigs are given as mean ± standard deviation. P-values, derived from one-way repeated measures ANOVAs are depicted as insets.

1.2 Supplementary Tables

	Day 0 (n = 5)	Day 21 (n = 5)
Sodium (mmol/l)	141.6±1.3	141.4±1.3
Potassium (mmol/l)	4.1±0.1	3.9±0.2
Calcium (mmol/l)	2.6±0.1	2.4±0
Magnesium (mmol/l)	0.7±0	0.7±0
Creatinine (mg/dl)	1.1±0	1.3±0.1
Urea (mg/dl)	16±3.4	25±1.1
hs-Troponin T (pg/ml)	4.9±1.3	8.3±1.9
LDH (U/l)	542.4±31.8	652.2±81.9
AST (U/l)	29.2±1.7	36.2±3.3
ALT (U/l)	42±1.5	52.4±3.3
AP (U/l)	152.2±15.1	143.6±8.8
GGT (U/l)	53±7.9	52±5.3
CRP (mg/l)	n.m.	n.m.
WBC (/nl)	16.2±3.1	14.2±2.7
RBC (/pl)	5.5±0.2	5.9±0.2
Hb (g/dl)	9.4±0.2	10±0.4
HCT (I/I)	0.3±0	0.3±0
MCV (fl)	51.8±1.2	51.6±1.1

MCH (pg)	16.8±0.4	17±0.3
MCHC (g/dl)	33±0.4	33.4±0.5
PLT (/nl)	384.8±29.8	363.2±37.3
INR (1/1)	1±0	1±0
aPTT (s)	26.5±11.1	9.8±0.5
NT-proBNP (ng/l)	n.m.	12±12

Table S1: Blood levels of LBBB pigs, followed up over 21 days. aPTT, prothrombin time test; ALT, alanine aminotransferase; AP, alkaline phosphatase; AST, aspartate aminotransferase; CRP, C-reactive protein; GGT, gamma-glutamyl transferase; Hb, hemoglobin; HCT, hematocrit; INR, international normalized ratio; LDH, lactate dehydrogenase; MCH, mean corpuscular hemoglobin; MCHC, mean corpuscular hemoglobin concentration; MCV, mean corpuscular volume; n.m., not measureable; PLT, platelets; RBC, red blood count; WBC, white blood count. Data of n = 5 pigs are given as mean ± SEM. No statistically significant differences from paired two-tailed Student’s t-tests followed by Bonferroni correction.

	Day 0 (n = 3)	Day 7 (n = 3)	Day 21 (n = 3)
Sodium (mmol/l)	139±1	140.3±1.2	139.7±0.7
Potassium (mmol/l)	3.8±0	4.1±0.2	3.7±0.1
Calcium (mmol/l)	2.5±0	2.5±0.1	2.4±0
Magnesium (mmol/l)	0.7±0	0.8±0	0.8±0
Creatinine (mg/dl)	1±0.1	1±0.1	1±0.1
Urea (mg/dl)	16±0	23.7±1.9	21±4.2
hs-Troponin T (pg/ml)	15.6±7.2	29±8.3	8.1±0.4
LDH (U/l)	532.3±32.7	674.7±98.9	620.7±82.2
AST (U/l)	32.3±6.2	44±5.6	47±4.2
ALT (U/l)	48.3±5	51.3±2.7	54±8.6
AP (U/l)	154.3±24.3	124.3±11	93.3±6.8
GGT (U/l)	43±3.5	40.7±5.2	162.3±120.4
Bilirubin (mg/dl)	n.m.	n.m.	n.m.
CRP (mg/l)	n.m.	n.m.	n.m.
WBC (/nl)	13.3±5.6	9.7±1.9	12.8±5.2
RBC (/pl)	5.3±0.3	19.6±14.2	5.5±0.2
Hb (g/dl)	9.3±0.7	8.8±0.6	8.9±0.1
HCT (l/l)	0.3±0	0.3±0	0.3±0
MCV (fl)	52.7±1.2	51.7±1.8	50.3±1.9

MCH (pg)	17.7±0.3	17.3±0.3	16.3±0.3
MCHC (g/dl)	33.3±0.9	33±0.6	32.7±0.3
PLT (/nl)	316.7±12.9	320.3±51.3	433.7±35.9
INR (1/1)	1.1±0	1±0	0.9±0
aPTT (s)	17.5±7.5	10.2±0.3	10.1±0.5
NT-proBNP (ng/l)	44±44	23.7±11.9	17±17

Table S2: Blood levels of LBBB pigs, that underwent CRT implantation. aPTT, prothrombin time test;ALT, alanine aminotransferase; AP, alkaline phosphatase; AST, aspartate aminotransferase; CRP, C-reactive protein; GGT, gamma-glutamyl transferase; Hb, hemoglobin; HCT, hematocrit; INR, international normalized ratio; LDH, lactate dehydrogenase; MCH, mean corpuscular hemoglobin; MCHC, mean corpuscular hemoglobin concentration; MCV, mean corpuscular volume; PLT, platelets; RBC, red blood count; WBC, white blood count. Data of n = 3 pigs are given as mean ± SEM. No statistically significant differences from one-way repeated measures ANOVAs.
